# Blood transfusion requirements for endoscopic sinonasal inverted papilloma resections

**DOI:** 10.1186/1916-0216-42-44

**Published:** 2013-07-16

**Authors:** Al-Rahim R Habib, Iain Hathorn, Vishnu S Sunkaraneni, Aviva Srubiski, Amin R Javer

**Affiliations:** 1 Division of Otolaryngology, University of British Columbia, St. Paul’s Sinus Centre, 1081 Burrard Street, Vancouver BC V6Z 1Y6, Canada

**Keywords:** Transfusion, Endoscopic sinus surgery, Computed tomography, Paranasal sinuses

## Abstract

**Background:**

Endoscopic resection of sinonasal Inverted Papilloma (SNIP) tumors has been shown to reduce intra-operative blood loss and recovery time compared to open approaches. The purpose of this study is to investigate the incidence and requirements of blood transfusion for endoscopic SNIP surgeries.

**Methods:**

An individual retrospective cohort study of endoscopic SNIP surgeries over a 10-year period was performed. Age, sex, pre-existing co-morbidity, use of anti-coagulants, tumor type and stage, time of surgery, estimated blood loss and the requirement for blood transfusion were recorded.

**Results:**

82 patients were included (57 males, 25 females). 4 (5%) Stage 1, 7 (8.5%) Stage 2, 62 (75.5%) Stage 3 and 9 (11%) Stage 4 SNIP tumors were identified according to the Krouse staging system. 3 (4%) patients required blood transfusion. 3 of the 9 (33%) Stage 4 tumors required blood transfusion. Stage 4 tumors were significantly associated with blood transfusion (p < 0.05). Higher staged tumors were associated with greater blood loss (p < 0.05) than lower staged cases. No other tumor stage required blood transfusion and no other pre-operative variable was associated with requirement for blood transfusion.

**Conclusion:**

Endoscopic SNIP resections rarely require blood transfusions. No pre-operative factor other than tumor stage is associated with the requirement for blood transfusion. We would therefore suggest that only Stage 4 SNIP tumors require pre-operative type and screen.

## Introduction

Sinonasal Inverted Papilloma (SNIP) is a benign epithelial growth occurring in the nose and paranasal sinuses [1]. These tumors commonly originate in the lateral nasal wall and/or middle meatus and can later extend to the surrounding paranasal sinuses [1]. Complete excision is the treatment of choice for these locally aggressive tumors with malignant potential [1-4]. Advances in endoscopic technology and instrumentation have improved visualization and surgical outcomes [1]. The evidence now suggests that endoscopic SNIP resection gives as good, if not better results than open approaches [5-8]. Endoscopic tumor resections are less invasive and associated with reduced surgical time, anaesthesia risk, shorter hospital stay, fewer intra-operative complications and lower recurrence rates when compared to the previous open craniofacial approach [3,4]. Nevertheless, excision of these tumors may cause significant bleeding and may occasionally require blood transfusion [4,9].

Blood transfusions restore and maintain circulating blood volume, correct anaemia and coagulation disorders [10]. However, allogeneic blood transfusions may pose a risk for transmissible diseases and adverse reactions [11]. In order to ensure accurate and compatible use of donor units, blood ordering practices such as type and screen testing and crossmatching have been utilized by transfusion laboratories [12]. The recipient’s blood is type and screened to determine ABO and Rh group and presence of RBC alloantibodies [12,13]. Crossmatching is utilized to determine patient and donor RBC, plasma and antibody compatibility [13]. This can be done electronically in some hospitals to save time and resources, as opposed to serological testing [14]. Although blood-ordering protocols for elective surgery are present to increase patient safety, practices of over-ordering units can develop [11,12]. This may occur due to habit, inexperienced staff or inadequate transfusion prediction models [11,12,15]. To assess the efficiency of routine blood ordering practices in elective surgery, several studies have observed the ratio of crossmatched to transfused patients, average number of units transfused for particular procedures and the transfusion probability coefficient (number of transfused patients/number of procedures) [9,12,15,16]. These studies have found that a tendency towards over-ordering of blood can consume blood bank resources and increase blood outdating [9,14-17].

It is unclear whether routine requisitions of blood are necessary for patients who undergo endoscopic SNIP surgery. To investigate the incidence and requirements for blood transfusion, we have conducted a retrospective chart review of endoscopic SNIP resections at our institution.

## Methods

A retrospective chart review of endoscopic SNIP resections performed at the St. Paul’s Sinus Centre (SPSC) between April 2000 to August 2010. This is a tertiary level sinus centre located at the University of British Columbia in Vancouver, Canada. The charts of those patients who had undergone computer assisted endoscopic resections were examined for patient demographic data, intra-operative details, type and screen ± crossmatch requisitions and the requirement for blood transfusion. Histopathological examination by the St. Paul’s Hospital Laboratory was used to determine tumor type from intra-operative specimens.

Patient demographic variables included age and sex. Pre-operative measurements consisted of the presence of pre-existing co-morbidities, previous sinus surgery, use of anti-coagulants, tumor stage, pre-operative hemoglobin and type and screen testing ± crossmatching. Patients taking anti-coagulation medication discontinued them 1 week prior to surgery. In addition, time of surgery, total blood loss and blood transfusion requirement were also documented. Blood loss estimates and time of surgery were ascertained from post-operative surgical notes and anaesthesia records. A fellowship trained Rhinologist (VSS) evaluated tumor stage pre-operatively from Computed Tomography (CT) scans and rigid nasal endoscopy. The Krouse staging system was used to evaluate all SNIP (Table 1) [18]. The senior author was present throughout all surgeries. The tumor was debulked piecemeal and the site of origin identified. The tumor was removed in its entirety and the bone underlying the origin was drilled to reduce any chance of recurrence. Biopsies were taken from all margins to ensure clearance. Hemostasis was achieved using suction diathermy for specific bleeding vessels and, on occasion, if significant general oozing was experienced topical epinephrine soaked neuropatties were used.

**Table 1:** Krouse staging system for inverted papilloma

	
T1	Tumor totally confined to the nasal cavity, without extension into the sinuses. The tumor can be localized to one wall or region of the nasal cavity, or can be bulky and extensive within the nasal cavity, but must not extend into the sinuses or into any extranasal compartment. There must be no concurrent malignancy.
T2	Tumor involving the ostiomeatal complex, and ethmoid sinuses, and/or the medial portion of the maxillary sinus, with or without involvement of the nasal cavity. There must be no concurrent malignancy.
T3	Tumor involving the lateral, inferior, superior, anterior, or posterior walls of the maxillary sinus, the sphenoid sinus, and/or the frontal sinus, with or without involvement of the medial portion of the maxillary sinus, the ethmoid sinuses, or the nasal cavity. There must be no concurrent malignancy.
T4	All tumors with an extranasal/extrasinus extension to involve adjacent, contiguous structures such as the orbit, the intracranial compartment, or the pterygomaxillary space. All tumors associated with malignancy.

The incidence of type and screen testing ± crossmatching was determined from the St Paul’s Transfusion Laboratory, through electronic and printed records. Patients who had undergone type and screen testing had their blood group identified and screened for the presence of Rh antibodies. The decision whether to type and screen a patient was made by the senior author along with the pre-assessment staff and anesthesiologist. If the surgical procedure had the potential to require blood transfusion, as judged by the senior author, crossmatching was completed with the required units reserved. All blood crossmatching was electronically completed through the Transfusion Laboratory. Similarly, blood transfusions were evaluated through Laboratory records and included total administered units. The amount of blood transfused was determined by evaluating the total amount of blood lost, the rate of blood loss, anesthesiology concern and the patient’s overall health.

The study outcomes were occurrence of “type & screen”, “cross-match” and “blood transfusion”. They were defined as binary variables in the analysis. For each outcome both univariate and multivariate logistic regression models were fitted to patient demographics and intra-operative variables with the occurrence of the outcome of interest as the dependent variable. A two-sided P value of less than 0.05 was considered to indicate statistical significance. Statistical analysis was conducted using Prism GraphPad Version 5.0.

## Results

### Total population

82 patients were identified as having undergone computer assisted endoscopic resections of SNIP in the study period, consisting of 57 (70%) males and 25 (30%) females. The mean age was 55 years. 23 (28%) patients had pre-existing co-morbidities, such as diabetes, hypertension, cardiovascular disease, liver disease and/or kidney disease. 6 (7%) patients were concurrently on a course of anti-coagulants, such as warfarin, acetylsalicylic acid or clopidogrel bisulfate. 45 (55%) of the total cases were primary endoscopic sinonasal IP resections. No patients were found to have pre-operative hemoglobin below 10 g/dL. No significant association was found between requiring blood transfusion and the use of anticoagulants, pre-existing co-morbidity, revision surgery or pre-operative hemoglobin (p > 0.05). The study population included 4 (5%) Krouse Stage 1, 7 (8.5%) Stage 2, 62 (75.5%) Stage 3 and 9 (11%) Stage 4 IP tumors (Table 2).

**Table 2:** Demographic and pre-operative details of total study population

***Total study population***	***n = 82***
*Age (years)*	*55*
*Males*	*57 (70%)*
*Females*	*25 (30%)*
*Pre-existing co-morbidity*	*23 (28%)*
*Use of anti-coagulants*	*6 (7%)*
*Primary sinus surgery*	*45 (55%)*
*Krouse Stage*	
*T4*	*9 (11%)*
*T3*	*62 (75.5%)*
*T2*	*7 (8.5%)*
*T1*	*4 (5%)*

### Transfusion

**Figure 1:**
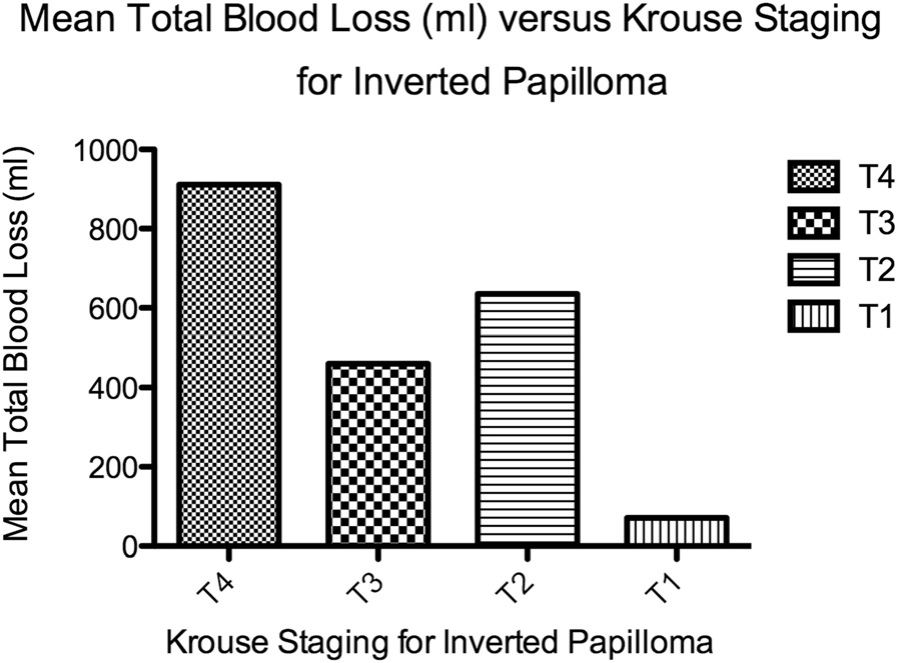
Mean total blood loss (ml) versus Krouse Staging for Inverted Papilloma.

3 (4%) patients required blood transfusion, all of whom had Krouse Stage 4 tumors. 3 of 9 (33%) Stage 4 tumors therefore required blood transfusion. Mean total blood loss for Stage 4 tumors (911 ml) was greater than Stage 3 (461 ml), Stage 2 (636 ml) and Stage 1 tumors (71 ml) (Figure 1). The three transfusion cases received one, two or four units of blood respectively. Higher staged tumors were associated with greater total blood loss than lower staged cases (p < 0.05). Stage 4 tumors were significantly associated with blood transfusion (p < 0.05). No significant association was found between tumor stage and time of surgery (p > 0.05). Overall, 1 in 27 SNIP cases were found to require blood transfusion. Higher staged tumors (Stage 3 and 4) that were found to have significant bleeding during surgery, the transfusion ratio increased to 1 in 24. For Krouse Stage 4, the transfusion ratio was 1 in 3.

### Type and screen testing

21 (26%) patients underwent type and screen testing. No association was found in patients’ type and screen tested and the presence of pre-existing co-morbidities (p = 0.32), use of anti-coagulants (p = 0.73), and pre-operative hemoglobin (p = 0.74). Of these, 5 (24%) were Stage 4, 14 (67%) Stage 3, and 2 (9%) were Stage 2 tumors. No Stage 1 tumors were type and screen tested (Table 3). Of all type and screened patients, only 3 (14%) required blood transfusion, all of which were Stage 4 tumors. 2 of 5 (40%) Krouse Stage 4 SNIPs that were screened did not require blood transfusion. Amongst the three cases requiring blood transfusion, 2 (66%) were not pre-screened. 18 (86%) of the type and screened cases did not require blood transfusion. The type and screen to transfusion ratio was 7:1 (Table 3).

**Table 3:** Krouse staging for cases type and screen tested and type and screen to transfusion ratio

***Type and screen***	***n = 21 (26%)***
*Krouse stage*	
*T4*	*5 (24%)*
*T3*	*14 (67%)*
*T2*	*2 (9%)*
*T1*	*0*
	
*Type and screen to transfusion ratio*	*7:1*

### Crossmatching

5 (6%) patients underwent blood crossmatch testing. Of these, 3 (60%) were Stage 4 and 2 (40%) were Stage 3 IP tumors. No Stage 1 or Stage 2 IP tumors were found to require blood crossmatch testing (Table 4). Of all blood crossmatched patients, 3 (60%) required blood transfusion. These cases only consisted of Stage 4 IP tumors. No other crossmatched tumor stage other than Stage 4 required blood transfusion. Stage 4 tumors requiring blood crossmatch testing had greater total blood loss (2300 ml) than those without crossmatch testing (217 ml). The crossmatch to transfusion ratio was 5:3 (Table 4). No significant association was found between the crossmatched patients and the presence of pre-existing co-morbidities (p = 0.78), use of anti-coagulants (p = 0.82), and pre-operative hemoglobin (p = 0.41).

**Table 4:** Krouse staging for crossmatched cases and crossmatch to transfusion ratio

***Crossmatch***	***n = 5 (6%)***
*Krouse stage*	
*T4*	*3 (60%)*
*T3*	*2 (40%)*
*T2*	*0*
*T1*	*0*
	
*Crossmatch to transfusion ratio*	*5:3*

### Expenditure

The estimated fees for each procedure were obtained from the Transfusion Laboratory and the Department of Finance to assess the cost of our current blood ordering protocol. The direct cost of type and screen testing was estimated to be $67.49. This included identification of patient blood type (ABO), presence of Rh groups and screening of circulating antibodies. Therefore, the total expenditure for all patients’ type and screen tested (n = 21) was approximately $1417.29. Similarly, the direct cost for electronic crossmatch testing was estimated to be $8.18. Patients’ blood was digitally matched to the donor blood bank to determine compatibility. Therefore, the total expenditure for all patients crossmatch tested (n = 5) was approximately $40.90. The combined cost of blood ordering procedures was $1458.19. All prices have been provided in Canadian dollars.

## Discussion

In this study we have observed that SNIP stage, operating time and total blood loss are the only measures that are associated with blood transfusion. It therefore appears that the only pre-operative variable identified in this study that can be used to predict who may require blood transfusion is tumor stage. This highlights the importance of a detailed pre-operative patient assessment to accurately stage the tumor. Advances in CT imaging and endoscopic visualization have allowed for better determination of the extent of SNIP tumors, allowing for improved assessment and surgical removal [19]. The amount of blood a patient would receive if transfused could not be predicted before surgery. Vascularity of the tumor or tumor bed, site of origin of the tumor and extent of resection during tumor removal were factors encountered intra-operatively that could not be accurately predicted pre-operatively. The study was performed in a tertiary referral sinus centre where the SNIP cases seen tend to be advanced, with 86% being Krouse Stage 3 or above. The incidence of blood transfusion may be even lower in non-tertiary centres where the cases are more likely to be of a lower Krouse stage.

Information gained from conducting an internal audit of surgical blood orders made at the St. Paul’s Sinus Centre can be used to establish precise guidelines for the allocation of blood resources. As described by Lin et al. (2006), for elective procedures where transfusion probability is less than five percent, pre-operative blood ordering can be disregarded [12]. We have found that the incidence of blood transfusion using the endoscopic approach for all sinonasal tumor resections is 4%. Approximately one out of seven patients type and screen tested was subsequently transfused (Figure 1). Those patients that were type and screened but not transfused had lower SNIP staging than those who required transfusion.

Our study suggests high SNIP tumor stage is an important factor that needs to be considered when deciding which patients require type and screen preoperatively. By determining SNIP stage from pre-operative CT imaging and endoscopic assessment, an efficient allocation of blood resources and surgical strategies can be made. Type and screen testing was approximately eight times more costly than electronic crossmatching, and therefore if unnecessary testing can be reduced a significant saving can be made. The overall incidence of blood transfusion was less than 5%. We therefore propose that routine type and screen is not required for all endoscopic resections of SNIP. However, blood transfusion was required in 33% of Stage 4 tumors, well above the 5% cut off suggested. We therefore recommend that type and screen testing should only be requested for Stage 4 SNIPs. If this protocol was followed in our series then all 9 Stage 4 SNIPs would have required type and screen testing at a cost of C$607.41, an overall saving of C$809.88. Introducing blood-ordering guidelines for surgical procedures can result in reduced costs, a lowering of the consumption of valuable blood bank resources as well as a decrease in blood outdating.

## Conclusion

Endoscopic SNIP resections rarely require blood transfusions. No pre-operative factor other than tumor stage was associated with the requirement for blood transfusion. We therefore recommend that only those patients with Stage 4 SNIP tumors require pre-operative type and screen.

## Competing interest

There are no financial disclosures or conflicts of interest to be reported.

## Authors’ contributions

ARH drafted the study protocol, performed chart reviews, participated in data analysis and drafted the manuscript. IH participated in designing research strategy, participated in data analysis and drafted the manuscript. VSS participated in designing research strategy, drafted the study protocol, participated in data analysis and drafted the manuscript. AS participated in designing research strategy, supervised data collection and drafted the manuscript. ARJ participated in designing research strategy, supervised data collection and analysis and finalized the manuscript. All authors read and approved this final manuscript.
